# Formate induces a metabolic switch in nucleotide and energy metabolism

**DOI:** 10.1038/s41419-020-2523-z

**Published:** 2020-05-04

**Authors:** Kristell Oizel, Jacqueline Tait-Mulder, Jorge Fernandez-de-Cossio-Diaz, Matthias Pietzke, Holly Brunton, Sergio Lilla, Sandeep Dhayade, Dimitri Athineos, Giovanny Rodriguez Blanco, David Sumpton, Gillian M. Mackay, Karen Blyth, Sara R. Zanivan, Johannes Meiser, Alexei Vazquez

**Affiliations:** 1 Cancer Research UK Beatson Institute, Glasgow, UK; 2 Center of Molecular Immunology, Havana, Cuba; 3 Institute of Cancer Sciences, University of Glasgow, Glasgow, UK; 4 Department of Oncology, Luxembourg Institute of Health, L-1526 Luxembourg, Luxembourg

**Keywords:** Metabolomics, Cancer metabolism, Cancer metabolism

## Abstract

Formate is a precursor for the de novo synthesis of purine and deoxythymidine nucleotides. Formate also interacts with energy metabolism by promoting the synthesis of adenine nucleotides. Here we use theoretical modelling together with metabolomics analysis to investigate the link between formate, nucleotide and energy metabolism. We uncover that endogenous or exogenous formate induces a metabolic switch from low to high adenine nucleotide levels, increasing the rate of glycolysis and repressing the AMPK activity. Formate also induces an increase in the pyrimidine precursor orotate and the urea cycle intermediate argininosuccinate, in agreement with the ATP-dependent activities of carbamoyl-phosphate and argininosuccinate synthetase. In vivo data for mouse and human cancers confirms the association between increased formate production, nucleotide and energy metabolism. Finally, the in vitro observations are recapitulated in mice following and intraperitoneal injection of formate. We conclude that formate is a potent regulator of purine, pyrimidine and energy metabolism.

## Introduction

Formate is a precursor of nucleotide synthesis and a potential growth-limiting factor^1,2^. Mammalian cells produce formate at rates exceeding the biosynthetic demand and excess formate is released from cells (formate overflow)^3,4^. Cells produce formate from the oxidation of the third carbon of serine using either a cytosolic or mitochondrial pathway^1,2^. Both pathways can sustain the one-carbon demands of cell proliferation, but the mitochondrial pathway is essential for formate overflow^4–6^. We have also shown that the catabolism of serine to formate is increased in tumours of genetically engineered mouse models of cancer resulting in an increase of serum formate levels^7^. Yet we do not understand what is the selective advantage of excess formate production by mitochondrial serine catabolism.

Mitochondrial serine hydromethyltransferase (SHMT2) provides methyl groups for the synthesis of taurinomethyluridine, which in turn is required for efficient mitochondrial protein synthesis^8^. Mitochondrial formate production also contributes to maintain low levels of cytosolic tetrahydrofolate^9^. Tetrahydrofolate is prone to oxidative damage and breakdown leading to the formation of toxic products^9,10^. In contrast, other folate species, such as 10-formyl-tetrahydrofolate, are more stable. Synthesis of 10-formyl-tetrahydrofolate from tetrahydrofolate and formate protects the cytosolic tetrahydrofolate pool from oxidative damage. However, there is no evidence that taurinomethyluridine synthesis or the protection of the tetrahydrofolate pool imply a one-carbon units demand comparable to the rate of mitochondrial serine catabolism to formate.

We hypothesize that formate overflow is associated with the highest metabolic demand of one-carbon units: purine synthesis. This hypothesis is counterintuitive because formate overflow is itself defined by excess formate production beyond the biosynthetic demand. However, the linear relationship implied by mass conservation (one-carbon produced = one-carbon consumed), needs to be put in the context of the non-linear kinetic relationships between metabolic rates and metabolite concentrations. In other words, we also hypothesize that formate overflow is rooted in a non-linear effect of enzyme kinetics. Here we provide evidence in support of this hypothesis.

## Materials and methods

### Chemicals

All cell culture material was obtained from Life Technologies and all the chemicals used were from Sigma-Aldrich unless stated otherwise.

### Cell lines and cultures

HAP1 cells were obtained from KJ Patel’s laboratory in the University of Cambridge. Cells were cultured in IMDM medium supplemented with 10% FBS and kept at 37 °C with 5% CO_2_. Cell counts and volumes were assessed using the Casy Technology (Innovatis). For Seahorse experiments, cells were mixed with Trypan Blue (50/50) and counted using the Countess optics and image automated cell counter (Life Technologies).

A lentiviral plasmid encoding SHMT2 CRISPR guide RNA was generated by cloning primers containing a SHMT2 guide RNA sequence^5^ into the lentiviral vector lentiCRISPR v2, a gift from Feng Zhang (Addgene plasmid # 52961; http://n2t.net/addgene:52961; RRID:Addgene_52961)^11^. HEK293T cells were transfected with above lentiCRISPR SHMT2 plasmid or control V2 plasmid together with helper plasmids pPAX2 and UVSVG (Addgene #8454 and #12260) using Lipofectamine 2000 (Life Technologies). Viral supernatant was harvested, filtered and incubated for 24 h on recipient cells lines (HCT116, MDA-MB-231, SKB3, T47D and MDA-MB-468) two consecutive times in the presence of 6 µg/µl polybrene (Sigma-Aldrich). Cells were selected for 10 days with 2 µg/µl puromycin (Sigma-Aldrich) to obtain a stable polyclonal population and knockout of SHMT2 expression was verified by western blot using a SHMT2 antibody (#12762) (Cell Signalling Technologies). All cells were cultured in in DMEM supplemented with 10% FBS and glutamine. SHMT2 deficient cells were cultured in the presence of HT supplement (Life Technologies).

### In vivo work

In vivo experiments were carried out in dedicated barriered facilities proactive in environmental enrichment under the EU Directive 2010 and Animal (Scientific Procedures) Act (HO licence numbers: 70/8645, 70/8468) with ethical review approval (University of Glasgow). Animals were cared for by trained and licensed individuals and humanely sacrificed using Schedule 1 methods. MMTV-PyMT (females) and APCmin (males & females) mice as previously described^7^. Wild-type female C57BL/6J mice (8 weeks) were purchased from Charles River.

In the formate bolus experiment, mice were randomized in individual groups (different time points and treatment arms). Mice were sacrificed at respective time points. Blood was immediately taken by cardiac puncture, transferred into Eppendorf tubes and centrifuged at 4 °C for 10 min at 13k G. The supernatant was transferred into new Eppendorf tubes and flash frozen in liquid nitrogen. Tissues were harvested in Eppendorf tubes and flash frozen in liquid nitrogen. Flash frozen tissue samples were blinded with random IDs and processed by a different person for metabolite extraction and analysis. After final data analysis IDs were uncovered. All tissues were processed frozen on dry ice. From each tissue 5–20 mg were balanced and transferred into Precellys CK14 tubes (Bertin Technologies, Montigny-le-Bretonnex, France). Tissues were dissolved in 20 mg/ml extraction solvent (Acetonitrile/MeOH/H_2_O (30/50/20)) and homogenized in a cooled Precellys 24 (Bertin Technologies, Montigny-le-Bretonnex, France) with 3 × 20 s at 7200 rpm and a 20-s break. Lysed tissue samples were transferred into Eppendorf tubes and centrifuged for 10 min at 4 °C. Supernatant was transferred into LC-MS vials for mass spec analysis.

### Intracellular formate quantification

HAP1 WT or SHMT2 KO cells were seeded in 6-well plates and stimulated with 1 mM formate (Sigma-Aldrich), for 24 h. One plate was used for counting, while cells in other plate were washed twice in ice-cold PBS and lysed in formate assay buffer at a ratio of 1,000,000 cells per 100 μl buffer. Lysates were transferred into Eppendorf tubes and centrifuged for 10 min at 14,000 rpm. Formate concentrations in supernatants were measured using a formate standard as a standard curve. Further proceedings were performed according to manufacters’ protocol of the Formate Assay Kit (Abcam, ab111748). In brief, formate assay buffer, enzyme mix, substrate mix were added to each standard and test sample in 96-well plates and incubated for 60 min at 37 °C. During the reaction, formate is oxidized to generate a product resulting in colour formation (*λ* = 450 nm) proportional to formate concentration. Readings were measured at OD450nm in a microplate reader.

### Metabolite quantification

Metabolite extraction and analysis was performed as previously described^12^. Briefly, cells were washed with PBS once and extracted with ice-cold extraction solvent (Acetonitrile/MeOH/H_2_O (30/50/20)), shaken for 5 min at 4 °C, transferred into Eppendorf tubes and centrifuged for 5 min at 18k G. The supernatant was transferred to LC-MS glass vials and kept at −80 °C until measurement. LC-MS analysis was performed as described previously using pHILIC chromatography and a Q-Exactive mass spectrometer (Thermo Fisher Scientific). Raw data analysis was performed using TraceFinder (Thermo Fisher Scientific) software. Peak areas were normalized to cell volume. Estimation of exchange rates and proliferation rates was done as described previously^4^.

### Protein, sample preparation

Cells were harvested by trypsinization, the pellet was washed twice in cold PBS then lyzed in 6 M Guanidinium HCL heated solution. Samples were boiled at 99 °C for 10 min then sonicated. Protein quantification was made using Bradford solution. Proteins were reduced with 10 mM DTT, for 30 min at 54 °C, and subsequently alkylated with 55 mM Iodoacetamide for 1 h at room temperature. Alkylated proteins were then submitted to a two-step digestion. First using Endoproteinase Lys-C (Alpha Laboratories) for 1 h at 35 °C, after which partial digests were further digested, with trypsin (Promega) overnight at 35 °C.

### Protein, MS analysis

Digested peptides were desalted using StageTip^13^ and separated on a nanoscale C18 reverse-phase liquid chromatography performed on an EASY-nLC 1200 (Thermo Scientific) coupled to an Orbitrap Q-Exactive HF mass spectrometer (Thermo Scientific). Elution was carried out using a binary gradient with buffer A: water and B: 80% acetonitrile in water, both containing 0.1% formic acid. Peptide mixtures were separated at 300 nl/min flow rate, using a 50 cm fused silica emitter (New Objective) packed in house with ReproSil-Pur C_18_-AQ, 1.9 μm resin (Dr Maisch GmbH). Packed emitter was kept at 50 °C by means of a column oven integrated into the nanoelectrospray ion source (Sonation). The gradient used started at 2% of buffer B (5 min), then increased to 16% over 185 min and then to 28% over 30 min. The eluting peptide solutions were electrosprayed into the mass spectrometer via a nanoelectrospray ion source (Sonation). An Active Background Ion Reduction Device (ABIRD) was used to decrease ambient contaminant signal level. Eluted peptides were analysed in the Orbitrap Q-Exactive HF. A full scan (FT-MS) was acquired at a target value of 3e6 ions with resolution *R* = 60,000 over mass range of 375–1500 amu. The top 15 most intense ions were selected for fragmentation using a maximum injection time of 50 ms or a target value of 5e4 ions.

### Protein, MS data analysis

The MS Raw files were processed with MaxQuant software^14^ version 1.5.5.1 and searched with Andromeda search engine^15^, querying UniProt^16^ *Homo sapiens* (09/07/2016; 92,939 entries). The database was searched requiring specificity for trypsin cleavage and allowing maximum two missed cleavages. Methionine oxidation and N-terminal acetylation were specified as variable modifications, and Cysteine carbamidomethylation as fixed modification. The peptide, protein and site false discovery rate (FDR) was set to 1%. Protein were quantified according to the label-free quantification algorithm available in MaxQuant^17^. MaxQuant output was further processed using Perseus software version 1.5.5.3^18^. The common reverse and contaminant hits (as defined in MaxQuant output) were removed. Only protein groups identified with at least one unique peptide were used for the analysis.

### Oxygen consumption rate

Cells were plated at 35,000 cells per well in a 96-well XF cell culture microplate (Seahorse Bioscience). Cells were equilibrated for 1 h at 37 °C in bicarbonate-free IMDM media (pH 7.3) with according treatments before any measurement. OCR and ECAR were measured three times every 9 min using a XFe96 Analyzer (Seahorse Bioscience) at a baseline and after addition of each drug. To assess the mitochondrial respiratory ability, oligomycin (1 μM), CCCP (1 μM), rotenone (1 μM) and antimycin A (1 μM) were injected subsequently. To assess glycolysis, oligomycin (1 μM) and 2-Deoxyglucose (50 mM) were added subsequently.

### Western blotting

HAP1 WT or SHMT2 KO cells were seeded in 60-mm dishes and stimulated with 1 mM formate (Sigma-Aldrich), 10 μM A769662 (Cayman Chemicals) or 1 μM AICAR (Sigma-Aldrich) as indicated. Cells were washed twice in ice-cold PBS and lysed in RIPA buffer (Thermo Scientific) containing cOmplete phosphatase and protease inhibitors (Sigma-Aldrich). Equal amount of proteins were separated by electrophoresis on 3–8% 1.0 mm Tris-Acetate NuPage gels (Thermo Scientific) and transferred to nitrocellulose using an Invitrogen XCell II Blot Module. Membranes were incubated overnight at 4 °C using the following primary antibodies: ACC phospho-Ser79 (#3661), total ACC (#3676), AMPK phospho-T172 (#2531), total AMPK (#2532) (Cell Signalling Technologies). Secondary antibodies were donkey anti-mouse 800CW and goat anti-rabbit IgG (H + L) Alexa Fluor 680 (Li-COR Biosciences and Thermofisher, respectively). Immunoblots were analysed and protein densities quantified using an Odyssey CLx imager and Image Studio Lite software (Li-COR Biosciences).

### Quantification and statistical analysis

Presented data are derived from three or more independent experiments, each with three technical replicates, unless specified. The average values for each independent experiment are indicated by the scatter symbols in the figures. The exceptions are the protein quantifications by mass spectrometry and the metabolomics data of the SHMT2 panel of deficient cell lines, were technical replicates were used. For two-groups comparisons the statistical significance was calculated with a Welch’s t test with two tails and unequal variance. The availability of one-carbon units was quantified by the index 0 (MFT-SHMT1), 1 (MFT, SHMT2), 2 (MFT + 1 mM Formate, SHMT2 + 1 mM Formate, WT). The protein changes were quantified by the slope of the log_2_ LFQ intensity vs the one-carbon availability index. The statistical significance of the slopes was estimated from 1 million permutations of the log_2_ LFQ intensities across the different cell lines/conditions. The enrichment of pathways for up and down regulated proteins was quantified by the gene set enrichment test^19^, using as input the slopes and the pathway annotations from The Molecular Signatures Database (MSigDB)^20^. The association of the remaining variables with the availability of one-carbon units was determined using the Spearman rank correlation coefficient (S) between the variable and the one-carbon availability index. The statistical significance of S was estimated from 1 million permutations of the variable values across the different conditions.

## Results

### Mathematical model

**Fig. 1:**
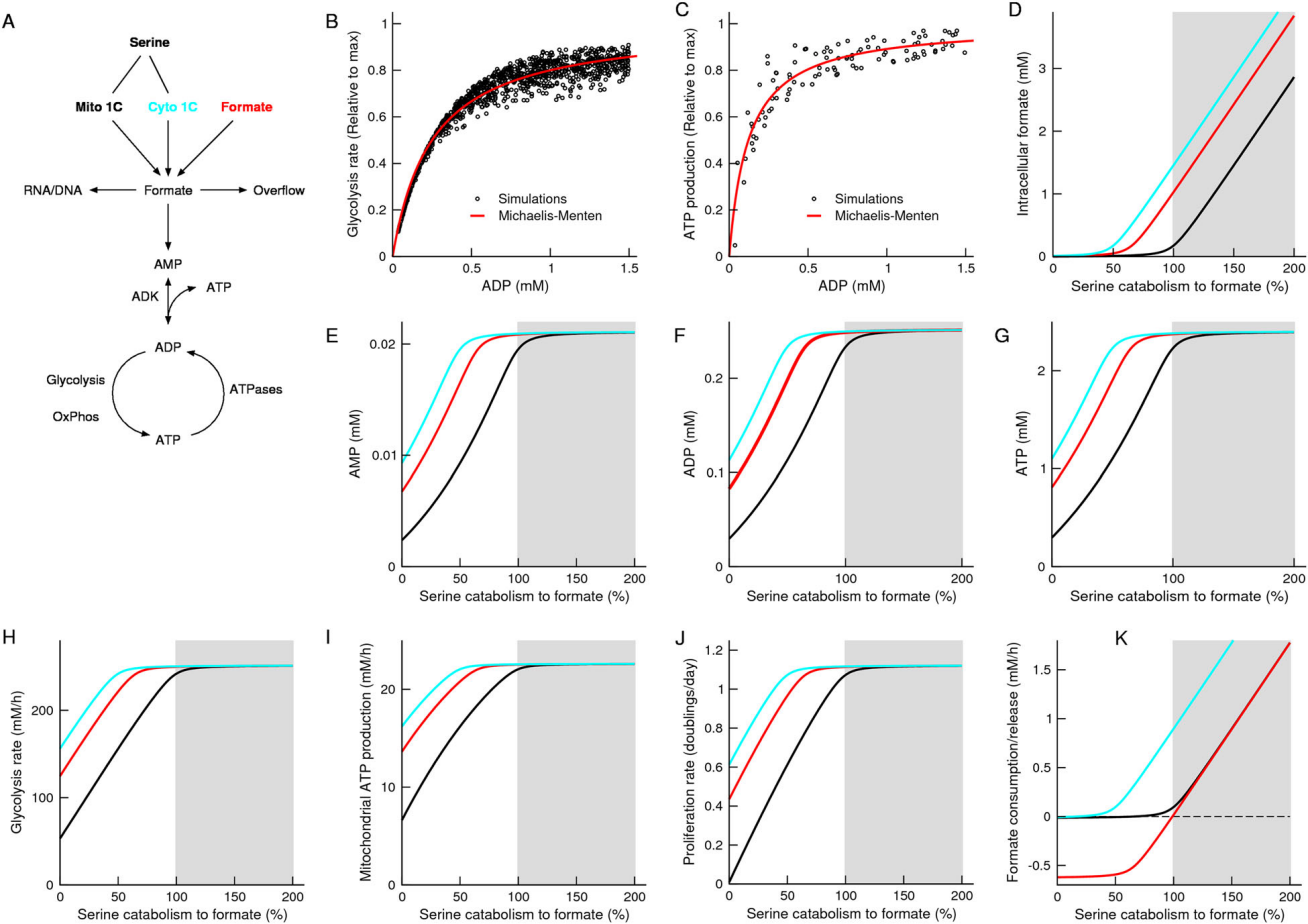
Theoretical model. **a** Graphical model linking formate and energy metabolism, including the contribution of the mitochondrial (Mito 1C) and cytosolic (Cyto 1C) one-carbon metabolism and extracellular formate (Formate). **b**, **c** Scatter plots of the simulated ADP phsophorylation rate by glycolysis and oxidative phosphorylation as a function of the ADP concentration. Each point represents a set of values for the different cofactors (ADP, ATP, NAD^+^, NADH). The line represents a fit to the Michaelis–Menten equation. **d**–**k** Model predictions with increasing the rate of formate production, with the addition of 0.02 mM extracellular formate (black), 1 mM extracellular formate (red) or 0.02 mM extracellular formate plus cytosolic one-carbon production (cyan). The grey background highlights the formate overflow state.

We analysed a simplified mathematical model where formate is produced from the mitochondrial one-carbon metabolism or consumed from the extracellular media (Fig. 1a, Supplementary text). The produced formate is released from cells or incorporated into adenine nucleotides. The free pools of adenine nucleotides (AMP, ADP and ATP) are established by the adenylate kinase equilibrium, ADP phosphorylation and ATPases activity.

Numerical simulations of kinetic models of glycolysis and oxidative phosphorylation indicate that the rates of ADP phosphorylation by glycolysis and oxidative phosphorylation follow effective Michaelis–Menten relationships with respect to the concentration of ADP (Fig. 1b, c). This observation is quite surprising given that both pathways have multiple reactions, with steps of ATP hydrolysis, ADP phosphorylation and oxidation/reduction reactions. These effective Michaelis–Menten models link energy and purine metabolism.

Next, we conducted numerical simulations of the full model of formate, purine and energy metabolism. The numerical simulations predict that, with increasing the rate of endogenous formate production, there is an increase in the concentration of formate and adenine nucleotides and the rates of glycolysis, oxidative phosphorylation and proliferation (Fig. 1d–j, black line). Furthermore, cells start releasing formate when the rate of serine catabolism to formate reaches the threshold rate defined by the purine synthesis rate (Fig. 1k, black line).

One-carbon units can be derived from extracellular formate and cytosolic metabolism as well. The effect of adding exogenous formate is to displace the prediction lines to the left (Fig. 1d–k, red line). To simulate the contribution of the cytosolic pathway to one-carbon unit production we added an additional flux of 10-formyl-tetrahydrofolate production, setting its rate to 50% the maximum activity of 10-formyl-tetrahydrofolate synthetase. The effect of the cytosolic pathway is similar to what observed for exogenous formate, it displaces the prediction lines to the left (Fig. 1d–k, cyan line).

In these simulations we assumed that the cell energy demand is coupled to an effective ATP consuming reaction with a Michalis-Menten dependency with the ATP levels. This assumption was motivated by the fact that some synthetases, like mammalian carbamoyl-phosphate synthetase (CAD), have a half-saturation constant on the mM ATP range^21^. An alternative hypothesis is that the proliferation rate is limited by the availability of deoxynucleotides, which in the case of adenines are derived from ADP. We have repeated the model simulations assuming that the proliferation rate follows a Michaelis–Menten relationship with the ADP levels and the outcome is the same (Fig. S1, red line). We have also repeated the simulations assuming a constant proliferation rate. In this case the transition from low to high purines is even more pronounced (Fig. S1, cyan line).

### In vitro model

**Fig. 2:**
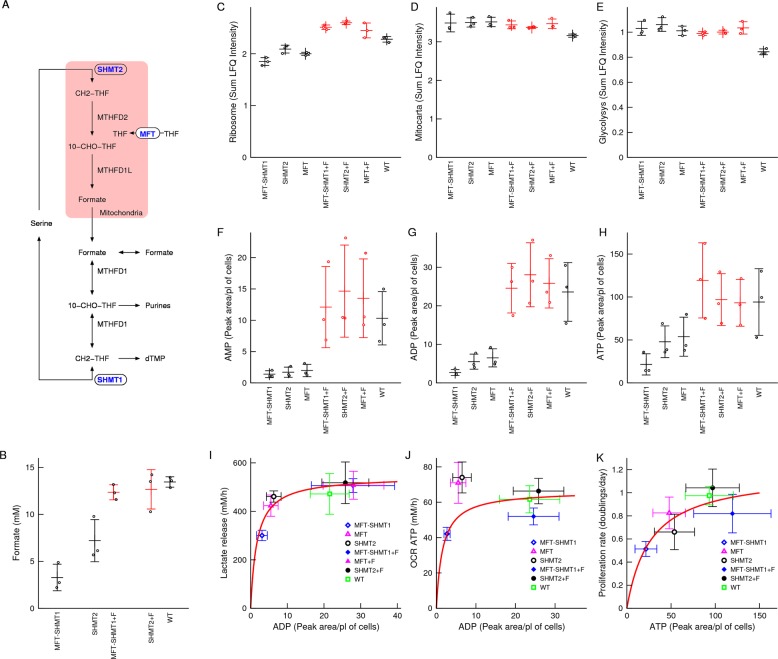
In vitro genetic model. **a** One-carbon metabolism pathway highlighting genes that were genetically inactivated (ovals). **b** Intracellular formate levels in the indicated cell lines. **c**–**e** Total protein mass associated with the indicated pathways. **f**–**h** Intracellular adenine metabolite levels. **i**–**k** Scatter plots of metabolic rates as a function of intracellular adenine nucleotide levels. The line represents a fit to a Michaelis–Menten equation. Notations: +F indicates 1 mM formate supplementation. Symbols represent independent experiments. Error bars represent the standard deviation.

To validate the theoretical predictions, we selected a panel of haploid HAP1 cell lines engineered for single knockout of SHMT2 or the mitochondrial folate transporter (MFT) and double knockout of MFT and cytosolic serine hydroxymethyltransferase (SHMT1) (Fig. 2a). The intracellular formate levels are the lowest in the MFT-SHMT1 double knockout cell line, intermediate in the SHMT2 single knockout cell line and maximum in the WT parental cell line (Fig. 2b). Furthermore, supplementation of sodium formate at a concentration of 1 mM increases the intracellular formate levels of the MFT-SHMT1 and SHMT2 cell lines to values similar to what is observed in the WT cells (Fig. 2b). Based on this data, we assigned the one-carbon units availability index 0 to MFT-SHMT1, 1 to MFT and SHMT2, and 2 to MFT + 1 mM Formate, SHMT2 + 1 mM Formate and WT.

We first characterized the proteome of these cell lines using mass spectrometry (Table S1). To identify protein level changes associated with the availability of one-carbon units we calculated the slope between the protein levels and the one-carbon availability index. We observed a significant increase in the levels of proteins belonging to the minichromosome maintenance protein complex (MCM) and of ribosomal proteins (Table S2). The increase in ribosomal proteins results in a gradual increase of the total ribosomal protein mass (Fig. 2c). In contrast, we observed just a trend towards decrease in the levels of mitochondrial and vacuole proteins (Table S2). The total proteome mass associated with proteins with annotated mitochondrial localization^22^ is approximately constant across the different cell lines (Fig. 2d). We did not observe any significant enrichment of genes associated with metabolic pathways, except for a trend of reduced levels of glycolysis and TCA proteins (Table S3). The total proteome mass associated with proteins in the KEGG glycolysis pathway is approximately constant across the different cell lines (Fig. 2e).

Next we performed a metabolic characterization. As predicted by the model, the levels of intracellular AMP, ADP and ATP increase from the knockout cell lines to the WT cell lines and when the knockout cell lines are supplemented with formate (Fig. 2f–h). In agreement with the behaviour suggested by the computational model of glycolysis (Fig. 1b), the rate of lactate release (a surrogate of glycolysis) approximately follows a Michaelis–Menten dependency with the intracellular ADP levels (Fig. 2i). In the case of oxidative phosphorylation the data deviates from a Michaelis–Menten law suggested by the computer simulations of mitochondrial oxidative phosphorylation (Fig. 2j). Finally, as assumed in the mathematical model, the proliferation rate approximately follows a Michaelis–Menten relationship with the intracellular ATP levels (Fig. 2k).

To test the metabolic switch beyond the HAP1 background, we generated a panel of SHMT2 deficient cell lines for the colorectal cancer cell line HCT116 and breast cancer cell lines MDA-MB-231, SKB3, T47D and MDA-MB-468. The parental cell lines exhibit formate overflow and the phenotype is lost upon genetic inactivation of SHMT2 (Fig. S2a). In the HCT116 and MDA-MB-231 background the SHMT2 deficiency causes a reduction in the adenine nucleotide levels (Fig. S2b–d), in agreement with our theoretical and HAP1 genetic models. However, this is not the case in the SKB3, T47D and MDA-MB-468 cell lines. Therefore, there are additional factors that modulate the control of the adenine nucleotide pools by mitochondrial formate production.

### Formate increases AICAR and reduces AMPK activity

**Fig. 3:**
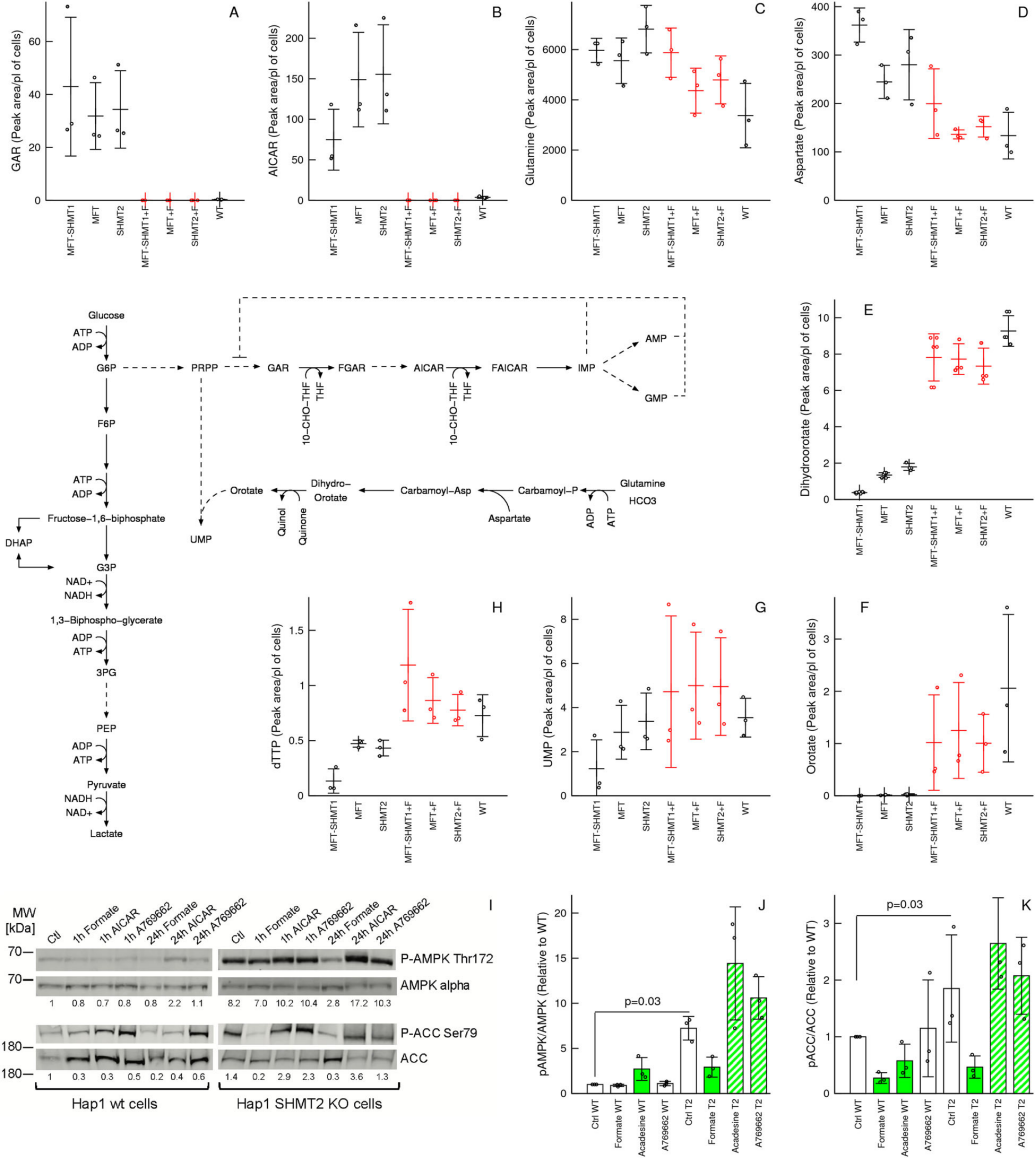
Formate incresses AICAR and suppresses the AMPK activity. **a**–**h** Metabolic changes associated with increasing the availability of one-carbon units. Only metabolites relevant for the discussion are reported. The full list of metabolites analysed can be found in Table S4. **i** Immunoblots of AMPK, phospho-AMPK, ACC and phospho-ACC (one representative experiment from three). **j**, **k** Quantification of immunoblots at the 24-h time point. Notations: +F denote 1 mM formate supplementation. Symbols represent independent experiments. Error bars represent the standard deviation. Solid bars indicate significant change (*p* < 0.05) and dashed bars trend (*p* < 0.1) relative to untreated cells of the same genetic background (two-sided, unequal variance, *T* test).

To uncover metabolic changes not anticipated by the mathematical model, we extended the correlation analysis between the intracellular metabolites and the one-carbon availability index (Table S4). We noted a significant negative association between the one-carbon availability index and the levels of purine precursors glycinamide ribonucleotide (GAR, *p* = 10^−6^) and 5-aminoimidazole-4-carboxamide ribonucleotide (AICAR, *p* = 5.4 × 10^−4^) (Fig. 2f vs Fig. 3a, b). The elevation of AICAR in cells deficient of mitochondrial one-carbon metabolism has been observed in other cell lines^5,23^. We also observe an elevation of AICAR in our panel of SHMT2 deficient cell lines (Fig. S1e). The effect being more pronounced in those cell lines where the SHMT2 deficiency is associated with a depletion of adenine nucleotides (Fig. S2b–d).

Given that both AICAR and AMP are AMPK activators^24^, the formate-dependent decrease of AICAR while increasing AMP results in conflicting signals to AMPK. To determine which signal dominates, we quantified the level of AMPK phosphorylation using phosphoantibodies specific for pAMPK-Thr172, a canonical AMPK site that is phosphorylated under energy stress^24^. We observed higher levels of pAMPK-Thr172/AMPK in SHMT2 deficient cells relative to WT cells (Fig. 3i, j). The noted changes in SHMT2 deficient cells are more pronounced than what was observed when treating WT cells with the AMPK activators acadesine (1 mM) or A769662 (10 μM). Finally, supplementation with 1 mM formate reduces pAMPK-Thr172/AMPK in SHMT2 deficient cells to levels between untreated SHMT2 deficient cells and WT cells, without much of an effect on WT cells.

Activated AMPK phosphorylates multiple proteins, including acetyl-CoA carboxylase (ACC) at serine 79 (Ser79)^24^. The changes of pACC-Ser79/ACC in SHMT2 deficient cells are quite similar to those observed for pAMPK-Thr172 /AMPK (Fig. 3j, k, T2 genetic background), indicating that in SHMT2 cells the level of ACC phosphorylation is either regulated by AMPK or by an upstream kinase targeting both AMPK and ACC. In contrast, the pattern of ACC phosphorylation in WT cells is different from that of AMPK phosphorylation. A clear example is the formate-dependent reduction of ACC phosphorylation in WT cells with no significant changes in AMPK phosphorylation, suggesting an AMPK-independent mechanism (Fig. 3j, k, WT genetic background).

Taken together these data indicate that AMPK activity is repressed by formate, either produced endogenously by mitochondrial one-carbon metabolism or supplemented exogenously.

### Formate increases pyrimidine nucleotides

The correlation analysis also revealed a positive association between the one-carbon availability and the levels of pyrimidine precursors dehydro-orotate and orotate and pyrimidines UMP and deoxythymidine triphosphate (dTTP) (Fig. 3e–h). Glutamine and aspartate, two precursors of pyrimidine synthesis, were rather depleted with increasing the availability of one-carbon units (Fig. 3c, d), excluding them as the cause of increased dehydro-orotate and orotate levels.

**Fig. 4:**
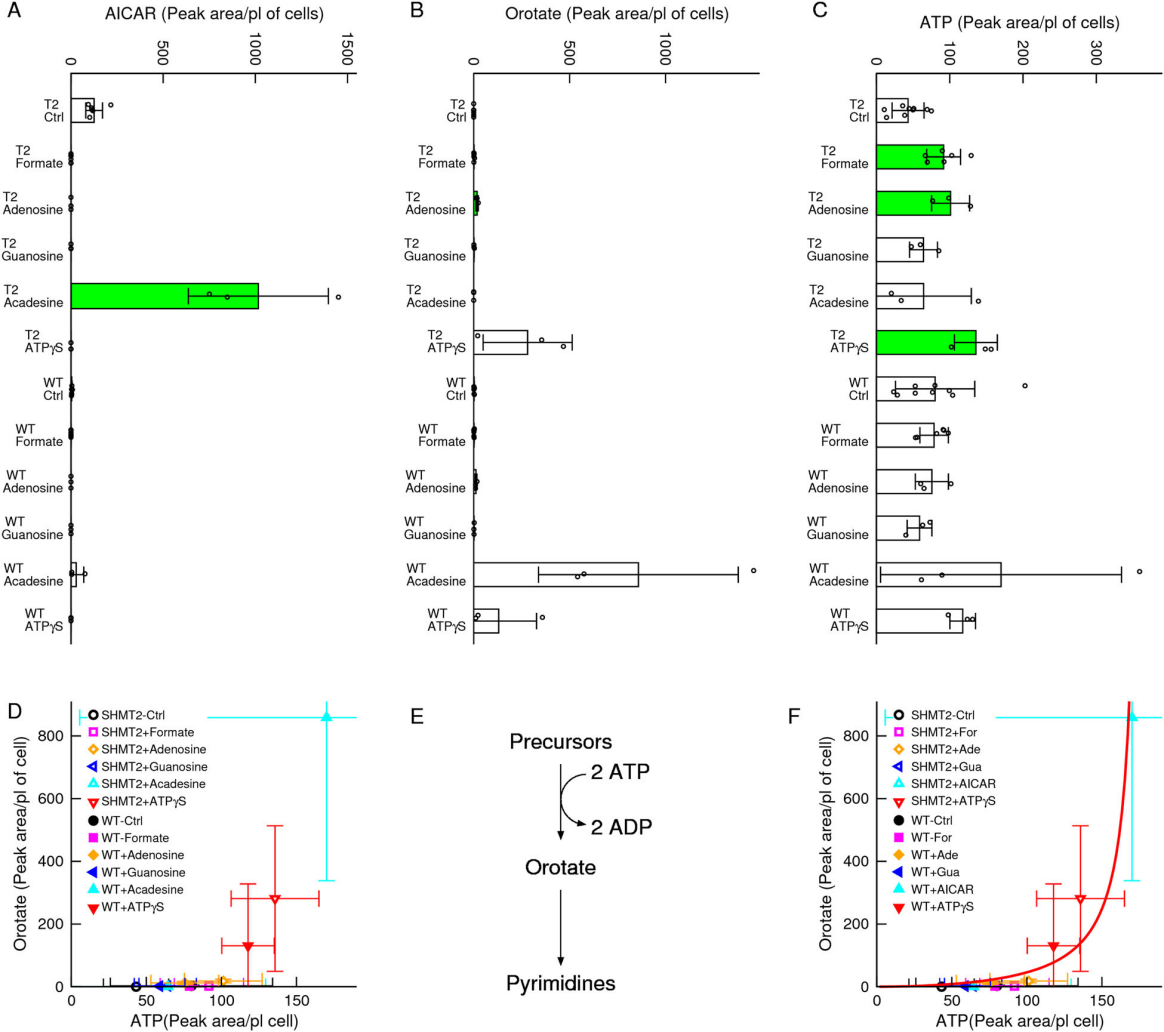
Evidence for an ATP-dependent increase in orotate. **a**–**c** Changes in AICAR, orotate and ATP following supplementation of 50 μM of purine metabolites to SHMT2 (T2) deficient and WT cells. **d** Scatter plot of orotate versus ATP. **e** Schematic model of orotate flux balance. **f** Fit of the theoretical model (line) to the scatter plot of orotate versus ATP. Notations: Symbols represent independent experiments. Error bars represent the standard deviation. Solid bars indicate significant change relative to untreated cells of the same genetic background (*p* < 0.05, two-sided, unequal variance, *T* test).

Acadesine, which is converted to intracellular AICAR after uptake and phosphorylation, was reported to increase orotate levels^25^. To follow this lead we performed purine nucleotides supplementation experiments and quantified intracellular metabolites. We found no association between endogenous AICAR and orotate levels (Fig. 4a, b). Since the first step of pyrimidine synthesis is catalysed by the ATP-dependent activity of carbamoyl-phosphate synthetase, we hypothesized that the observed changes in orotate levels are determined by changes in ATP levels. Indeed, there is a better association between the intracellular levels of orotate and ATP, than between orotate and AICAR (Fig. 4a–c). This association is more evident in a scatter plot of orotate versus ATP levels (Fig. 4d). These data are explained by a theoretical model of orotate metabolism (Fig. 4e, Supplementary Text). When the maximum orotate production rate exceeds that of turnover the model predicts a steep increase in orotate levels as the levels of ATP approach a limiting value, as observed (Fig. 4f).

The changes in dehydro-orotate and orotate levels are recapitulated in the panel of SHMT2 deficient cell lines (Fig. S2f, g). In the HCT116, MDA-MB-231 and SKB3 backgrounds the SHMT2 deficiency causes a drop in ATP levels that is accompanied by a drop in dehydro-orotate and orotate levels. In contrast, in the MDA-MB-468 and T47D cell lines, where the SHMT2 deficiency does not decrease the ATP levels, there are no appreciable changes in the dehydro-orotate and orotate levels.

### Formate supplementation

**Fig. 5:**
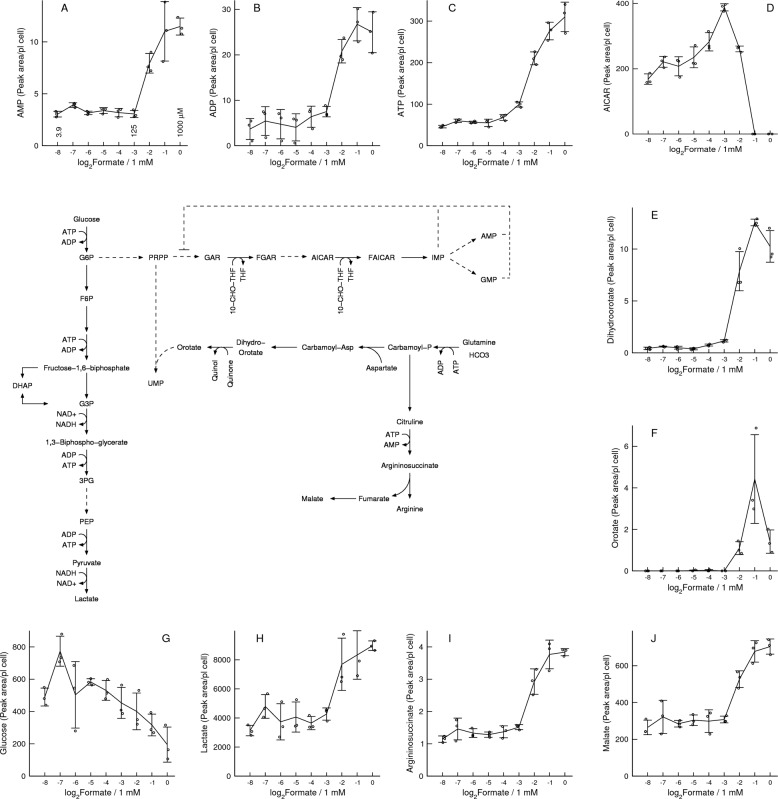
In vitro model of formate supplementation. **a**–**j** Metabolic changes associated with formate supplementation to the MFT-SHMT1 cell line, using twofold dilutions: 1 mM (0), 0.5 mM (−1), 0.25 mM (−2), 0.125 mM (−3), 0.0625 mM (−4), 0.031255) mM (−5), 0.015625 mM (−6), 0.0078125 mM (−7) and 0.00390625 mM (−8). Notations: Symbols represent independent experiments. Error bars represent the standard deviation.

To provide additional evidence, we have titrated the amount of formate supplemented to the MFT-SHMT1 deficient cell line and quantified intracellular metabolites (Fig. 5a–j). Formate supplementation induced a dramatic difference in metabolite concentrations at a formate concentration of about 100 μM. Below this concentration the adenine nucleotide levels are low and approximately constant (Fig. 5a–c), increasing twofold or higher at formate concentrations of 500 μM or 1 mM. At low supplemented formate concentration (below 100 μM), AICAR increases with increasing the concentration of supplemented formate, then drops down to undetectable levels at the formate concentrations of 500 μM or 1 mM (Fig. 5d). Dehydro-orotate and orotate also exhibit a sharp increase above a supplemented formate concentration of 100 μM (Fig. 5e, f). Finally, there is a gradual decrease of the intracellular glucose concentration with increased concentration of supplemented formate (Fig. 5g). In contrast, the intracellular lactate levels exhibit a switch-like behaviour, with a sharp increase above a supplemented formate concentration of 100 μM (Fig. 5h). The switch-like increase in lactate levels can be explained by the association between the rate of glycolysis and the ADP levels and the switch-like increase of ADP levels induced by formate (Fig. 5b).

To search for additional metabolites that could be modulated by ATP levels we calculated the spearman correlation coefficient between ATP and intracellular metabolite levels (Table S6). As anticipated by the results described above, the top associations included a positive correlation with purines and pyrimidines and a negative association with the purine synthesis intermediate metabolites (GAR, SAICAR, AICAR). We also noted a positive correlation between ATP levels and the levels of argininosuccinate (Fig. 5i). Argininosuccinate synthetase is an ATP driven enzyme that, as carbamoyl-phosphate synthetase, exhibits cooperativity for ATP^26^. A kinetic study of yeast argininosuccinate synthetase indicates that the enzyme kinetics is characterized by a sigmoidal dependency with respect to the concentration of ATP, with a Hill coefficient of 2. Therefore, similarly to orotate, the formate-dependent increase of argininosuccinate can be explained by the formate-dependent increase of ATP and the ATP-dependent activity of arginonosuccinate synthetase. We also noted that malate is increased following formate supplementation (Fig. 5j). These changes are consistent with the fact that fumarate is a by-product of both argininosuccinate turnover and purine synthesis and that fumarate is converted to malate by fumarate hydratase. The formate-dependent induction of argininosuccinate and malate is not recapitulated when comparing the panel of SHMT2 deficient cell lines with their parental cell lines (Fig. S2h, i).

### Pharmacological inhibition

Going in the opposite direction, we tested the formate-dependent metabolic switch in the context of pharmacological inhibition using the serine hydroxymethyltransfarece inhibitor SHIN1^27^. The data are an almost specular image of what is observed in the formate supplementation experiments (Fig. S3a–j). From this data we can conclude that inhibition of serine hydroxymethyltransfarase activity causes a systemic inhibition of cell metabolism that is mediated by the formate-dependent metabolic switch uncovered here.

### In vivo validation in mouse models of cancer

**Fig. 6:**
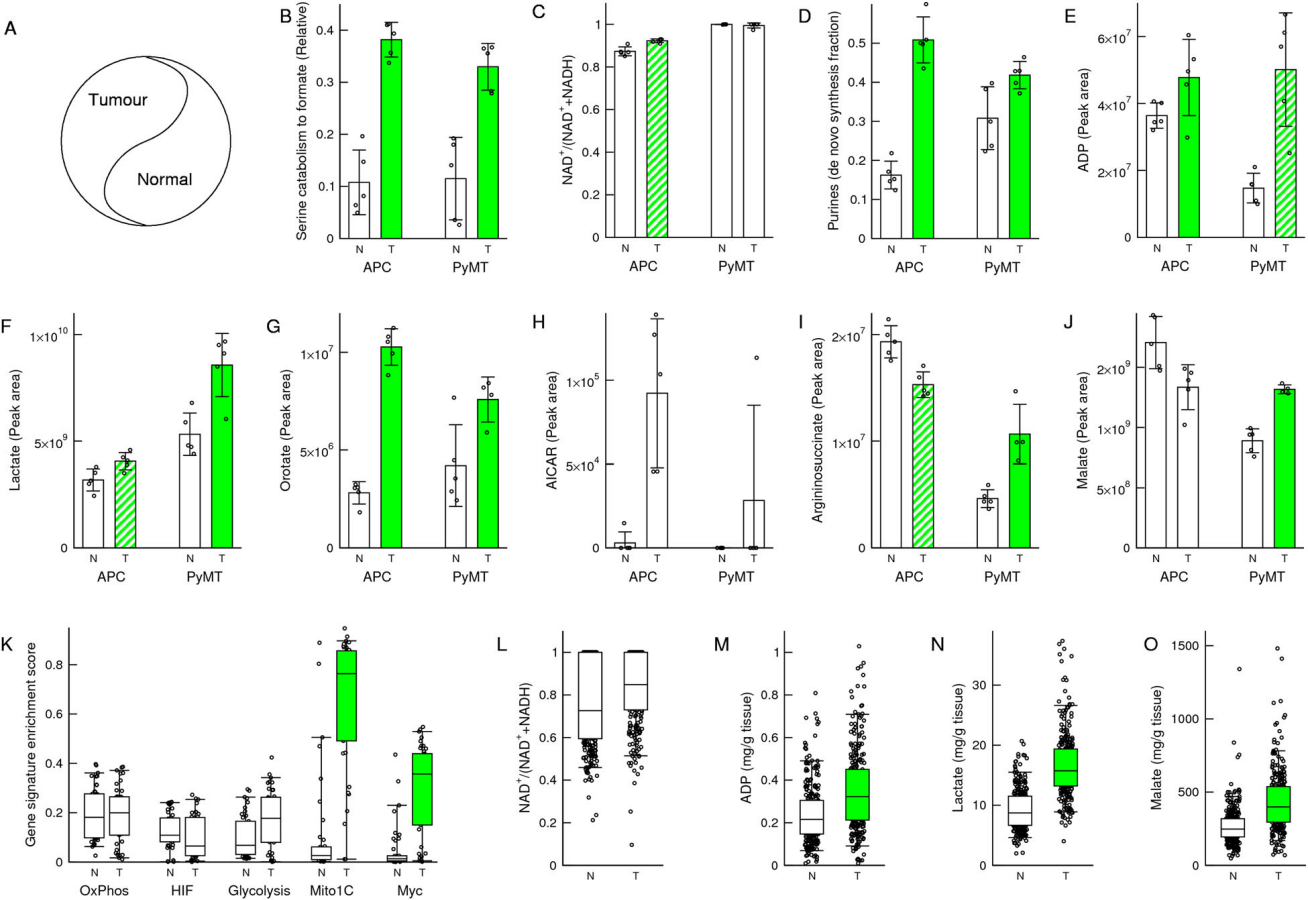
In vivo validation in cancer models. **a** Schematic representation of the normal and tumour tissue composition. **b**–**j** Metabolic features of transformed (T) and adjacent normal (N) tissues of the APC^min/+^ and PyMT mouse models of colorectal adenomas and breast adenocarcinomas. Notations: Each symbol represents a different mouse. Error bars indicate standard deviation. **k**–**o** Gene signature enrichment scores (**k**) and metabolic features (**l**–**o**) of human colorectal tumours and adjacent normal tissue. Notations: The error bars indicate 90% confidence intervals, the boxes 50% confidence intervals and horizontal line the median. Symbols outside the boxes represent individual samples. Green solid or dashed bars indicate significance increase (*p* < 0.05) or trend (*p* < 0.1) in transformed tissue relative to normal (two-sided, unequal variance, *T* test).

To provide an in vivo validation we analysed differences between tumours and the adjacent normal tissues (Fig. 6a). As previously shown, the relative rate of serine catabolism to formate is increased in the transformed tissues relative to the adjacent normal tissues, in the APC^min/+^ mouse model of colorectal adenomas and the PyMT model of breast adenocarcinoma^7^ (Fig. 6b). We have also shown that the transformed tissues have a high NAD^+^/(NAD^+^ + NADH) ratio that is similar than the adjacent normal tissue^7^ (Fig. 6c). The latter suggests that the tumour tissues have a similar redox status than the adjacent normal tissue. We have re-analysed the LC-MS data to extract the quantifications of relevant metabolites. The fraction of de novo synthesized purines, a surrogate of the purine synthesis rate, is significantly higher in the tumour tissues than in the adjacent normal tissues (Fig. 6d). The levels of ADP are increased in the transformed tissues relative to the adjacent normal tissues (Fig. 6e, trend in the APC^min/+^ model and significant in the PyMT model). Furthermore, the levels of lactate are increased in the transformed tissues relative to the adjacent normal tissues (Fig. 6f, trend in the APC^min/+^ and significant in PyMT models). Although these associations are not causal proof, they are consistent with our mechanistic model of increased formate production purine synthesis, ADP levels and lactate levels. In agreement with the in vitro models, there is also a significant increase in the levels of orotate in the tumour tissue relative to the adjacent normal tissue (Fig. 6g).

The AICAR levels are increased in the tumour tissue relative to the normal tissue (Fig. 6h). Based on our in vitro data this change would be the expectation if the transition happens from low to intermediate formate availability. In the genetic in vitro model, AICAR increases from the MFT-SHMT1 deficient to the SHMT2 or MFT deficient cell lines, then dropping in the WT cells (Fig. 3b). In the in vitro model of formate supplementation, AICAR increases when the MFT-SHMT1 deficient cells are supplemented with formate up to a concentration 100 μM, then dropping at 1 mM supplemented formate (Fig. 5d). We note that aspartate and glutamine, which are required as cofactors both upstream and downstream of AICAR, exhibit significantly higher levels in the tumour tissue relative to the normal tissue, while glycine is not significantly different (Fig. S4a–c). This increase in aspartate and glutamine levels in the tumour tissue may also contribute to the increased AICAR levels.

Finally, the levels of argininosuccinate and malate are significantly increased in tumour tissue of the PyMT model, but not in the APC^min/+^ model (Fig. 6i, j). A similar discrepancy was observed in our in vitro models. The HAP1 cells manifest a SHMT2-dependent elevation of the intracellular argininosuccinate levels, but this is not the case for the other cell lines tested. This discrepancy is anticipated by our theoretical analysis (Supplementary Text), depending on the relative maximum activity of argininosuccinate synthesis and turnover. In turn, the lack of significant changes of malate in the APC^min/+^ model could be the consequence of lack of changes in argininosuccinate, which is turned over to fumarate and subsequently to malate (Fig. 5, pathway inset).

### In vivo validation in human colorectal cancer

Recently Satoh et al. have shown that colorectal cancers are characterized by a global metabolic reprogramming induced by Myc^28^. Since MYC activates the transcription of mitochondrial one-carbon metabolism genes^3,29^, we hypothesized that MYC-driven colorectal cancers should manifest the formate-dependent metabolic switch. To test this hypothesis, we first performed a gene signature analysis using the reported gene expression array data for 41 colorectal tumour samples and 39 normal colorectal samples^28^. Using gene set enrichment analysis^19^ we quantified the enrichment of relevant gene signatures in the different samples (gene signature enrichment score). There are no significant differences in the enrichment scores for gene signatures of oxidative phosphorylation, HIF1α targets and glycolysis (Fig. 6k). In contrast, there is a significant increase of the mitochondrial one-carbon metabolism enrichment score signature in the tumour relative to the normal samples (Fig. 6k). The latter is also consistent with an increase of the enrichment score of a MYC targets signature in the tumour relative to the normal colorectal samples (Fig. 6k).

Next, we analysed reported metabolomic data from 275 normal and 275 tumour samples^28^. The tumour tissues exhibit a high NAD^+^/(NAD^+^ + NADH) ratio that is not significantly different from that of the normal tissues (Fig. 6l). That together with the HIF targets signature evidence suggest that the MYC-driven colorectal tumours are of oxidative nature and that their oxidative status is not significantly different from that of normal tissues. In contrast, there is a significant increase in the levels of ADP, lactate and malate in the tumour samples relative to the normal tissues (Fig. 6m–o). Although these associations are not causal prove, they are consistent with what predicted by the formate-dependent metabolic switch.

### In vivo validation following a formate bolus

**Fig. 7:**
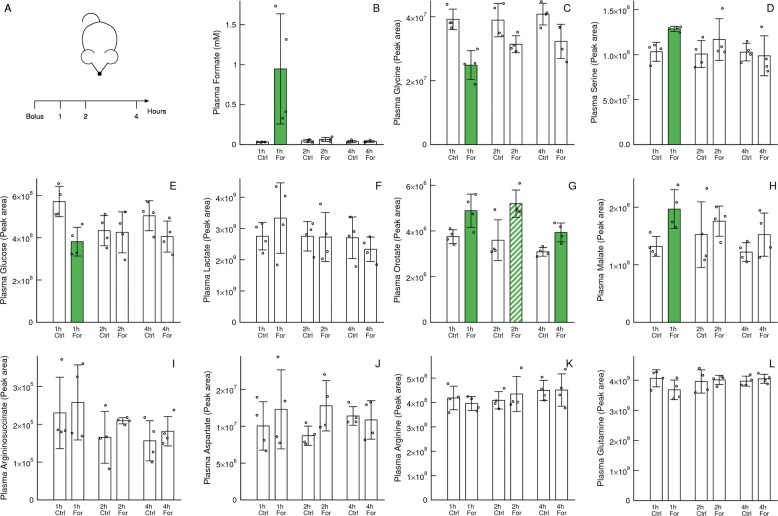
In vivo formate bolus. **a** Experiment design. A bolus of 13C-formate (For) or vehicle (Ctrl) was injected intraperitoneally to C57BL/6J mice. **b**–**l** Plasma metabolite levels after administration of the formate bolus or vehicle. Samples were collected from different mice at the indicated time intervals after the bolus injection. Notation: Each symbol represents a different mouse. Error bars indicate standard deviation. Solid bars indicate significant change or trend relative to control (*p* < 0.05 and *p* < 0.1, two-sided, unequal variance, *T* test).

To provide a direct in vivo validation of the formate-dependent metabolic switch we intraperitoneally administered a bolus of formate or vehicle to C57BL/6J mice fasted overnight (Fig. 7a). Different mice were used to collect plasma samples at 1, 2 and 4 h after the bolus injection. Plasma formate reached between 500 μM to 1 mM levels 1 h after the bolus injection, going down to μM levels 2 h after the bolus injection (Fig. 7b). At 1 h there is a significant depletion of plasma glycine (Fig. 7c) and a significant increase of plasma serine (Fig. 7d), which are consistent with the reverse activity of liver serine hydroxymethyltransferase and the administration of excess formate.

At 1 h, when the observed formate concentration was highest, there is a significant depletion of plasma glucose (Fig. 7e) and a non-significant trend towards increased plasma lactate (Fig. 7f). These changes are consistent with the formate-dependent induction of glycolysis. The lack of significant changes in plasma lactate could be due to lactate oxidation at the tissues where glucose metabolism is increased.

As observed in our in vitro models, the formate bolus induces a significant increase of plasma orotate and malate levels at the 1-h time point (Fig. 7g, h). In the case of orotate the significant increase persists 4 h after bolus injection. Since all other significant changes are absent at the 2- and 4-h time points, the simplest explanation is that the orotate turnover is slow, taking a long time to come back to control levels. In contrast, the levels of argininosuccinate does not change significantly at any time point (Fig. 7i). Finally, there are no significant changes in the levels of other amino acids implicated in purine, pyrimidine and argininosuccinate metabolism (aspartate, arginine and glutamine, Fig. 7j–l).

Therefore, a bolus of formate causes changes at the level of whole-body metabolism that are similar to what observed in our in vitro models.

## Discussion

Our analysis indicates that formate induces a metabolic switch in purine, pyrimidine and energy metabolism. The increase in purine nucleotides was expected given that formate is a precursor of de novo purine synthesis^1,2^. The formate-dependent induction of the increase in the pyrimidine precursor orotate can be explained, at theoretical level, by the ATP-dependent activity of carbamoyl-phosphate synthetase. Finally, provided that the levels of glycolytic enzymes remain constant, the formate-dependent increase in ADP levels is associated with an increase in the rate of glycolysis and intracellular lactate levels.

In contrast, formate deficiency causes a dramatic increase in AICAR levels and induces AMPK activity. The activation of AMPK in formate deficient cells is likely mediated by the dramatic increase in AICAR levels, the reduction in purine levels, or a combination of both. A similar phenotype is achieved with purine synthesis inhibitors. This has been shown for antifolates such as pemetrexed and methotrexate^30–33^ and for a dimerization inhibitor of AICAR formyltransferase as well^34^.

Further work is required to determine the relevance of the formate-dependent metabolic switch in the context of embryonic development, cancer and immune system metabolism. Homozygous deletion of *MTHFD1L*, whose gene product contribute to the mitochondria formate production, is embryonic lethal and it can be rescued by formate supplementation^35^. The formate-dependent metabolic switch could explain the requirement of mitochondrial one-carbon metabolism during embryonic development. There is also evidence for a partial dependency on mitochondrial one-carbon metabolism for cancer growth. Deprivation of serine in the diet delays tumour growth in genetic mouse models of cancer^36^. Suppression of mitochondrial one-carbon metabolism genes reduces growth in xenograft models of cancer^5,37^. Further work is required to investigate whether the reduction in cancer growth is determined by a reduction in nucleotide synthesis, energy metabolism or a contribution of both. Mitochondrial serine catabolism to formate is also essential for T-cell expansion^38,39^. Defective respiration and mitochondrial one-carbon metabolism contribute to a reduction in T-cell activation during aging of mice^40^. This evidence together with the long known role of aerobic glycolysis in T-cell activation^41,42^ suggests a role for the proposed formate link between respiration and glycolysis during T-cells activation.

## Supplementary information

Supplementary figure captions

Figure S1

Figure S2

Figure S3

Figure S4

Supplementary text

Supplementary tables

Supplementary Octave script

## Data Availability

The MS protein quantifications and the associated pathway analysis data in Fig. 2 are reported in Tables S1–S3. The metabolites considered for the intracellular metabolomics analysis and the association between their levels and the one-carbon availability index are reported in Table S4.
