# Supplementary material for: Formate induces a metabolic switch in nucleotide and energy metabolism
Source: Cell Death Dis. 2020 May 4;11(5):310. doi: 10.1038/s41419-020-2523-z (PMC7198490; doi:10.1038/s41419-020-2523-z)
Supplement: Supplementary file 1 — Supplementary figure captions [file 41419_2020_2523_MOESM1_ESM.docx]

**Figure S1.**

**Test of different proliferation rate models.** Theoretical model simulations illustrating that the increase in purine levels is independent on the proliferation rate assumptions. The black line represents a model where the proliferation rate follows a Michaelis-Menten relationship with the levels of ATP, as shown in the main text. The red line represents a model where the proliferation rate follows a Michaelis-Menten relationship with the levels of ADP. The cyan line represents a model where the proliferation rate is constant. In the latter case the supply of one-carbon units needs to be above the one-carbon demand associated with the synthesis of nucleotides for the specified constant proliferation rate.

**Figure S2.**

**Metabolic changes in a panel of SHMT2 deficient cell lines.** Levels of intracellular metabolites in different cell lines with inactivated SHMT2 (SHMT2 KO) and their parental cell lines. The data corresponds to a single experiment with 3 samples per cell line.

**Figure S3.**

***In vitro* model of pharmacological formate deprivation.** A-J) Metabolic changes associated with treatment of WT HAP1 cells with the serine hydroxymethyltransferase inhibitor SHIN1, using 2-fold dilutions: 10 μM (0), 5 μM (-1), 2.5 μM (-2), 1.25 μM (-3), 0.625 μM (-4), 0.31255 μM (-5), 0.15625 μM (-6), 0.078125 μM (-7) and 0.0390625 μM (-8). Notations: Symbols represent independent experiments. Error bars represent the standard deviation.

**Figure S4.**

***In vivo* validation in cancer models (expanding from Fig. 6).** Levels of purine precursors in transformed (T) and adjacent normal (N) tissues of the APC^min/+^ and PyMT mouse models of colorectal adenomas and breast adenocarcinomas. Notations: Symbols represent different mice. Error bars represent the standard deviation. Solid bars indicate significant change (p<0.05) relative to untreated cells of the same genetic background.
