# Supplementary figures and images for: Formate induces a metabolic switch in nucleotide and energy metabolism
Source: Cell Death Dis. 2020 May 4;11(5):310. doi: 10.1038/s41419-020-2523-z (PMC7198490; doi:10.1038/s41419-020-2523-z)

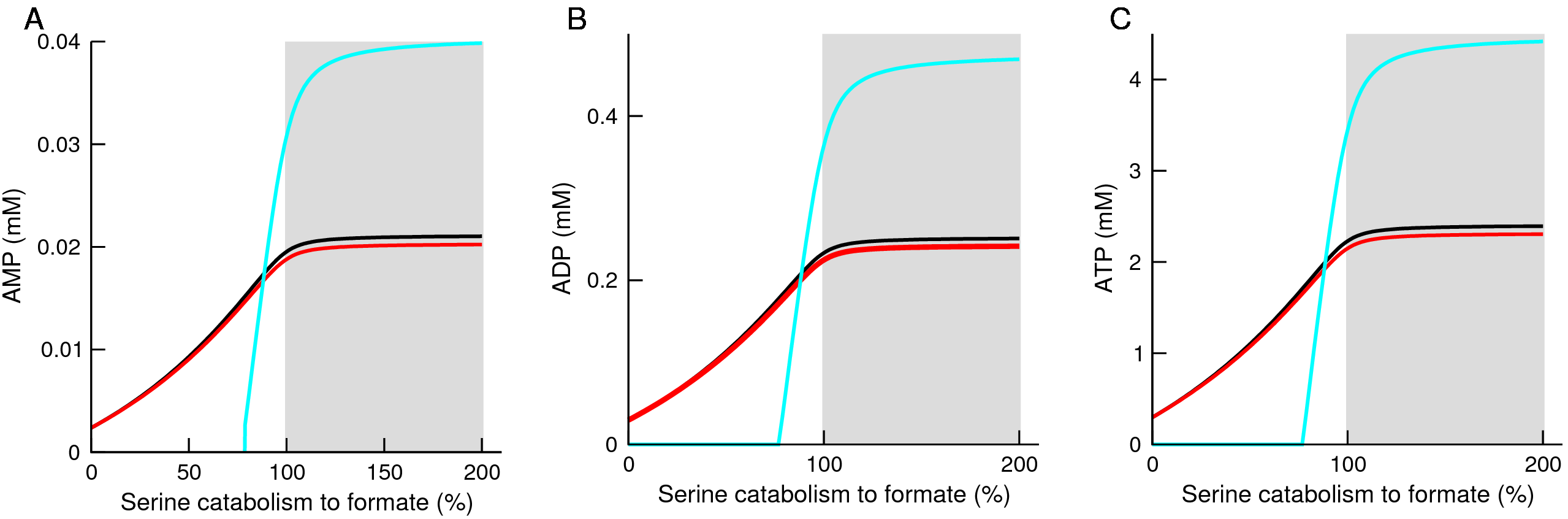

Supplement: Supplementary file 2 — Figure S1 [file 41419_2020_2523_MOESM2_ESM.tif]

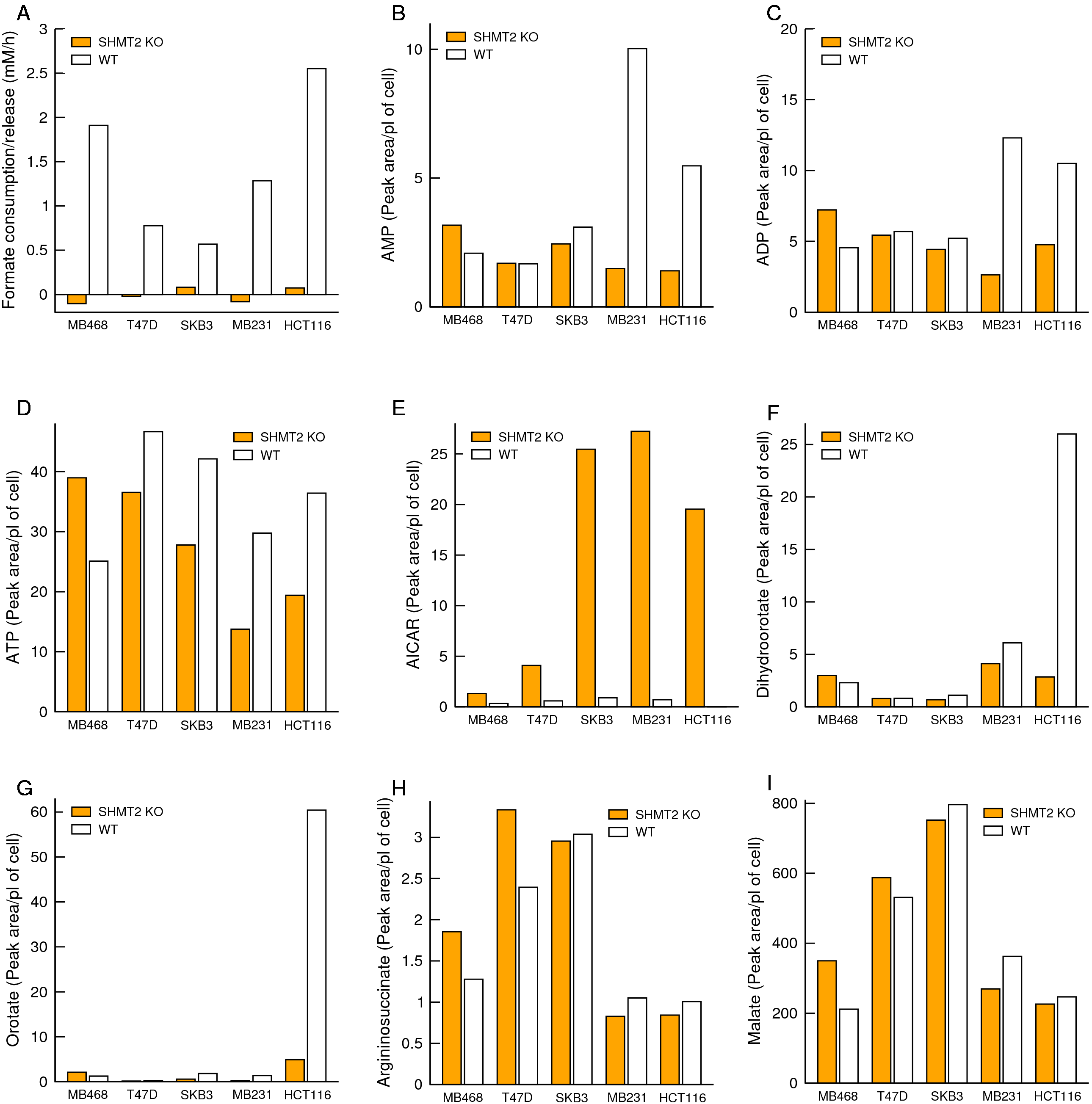

Supplement: Supplementary file 3 — Figure S2 [file 41419_2020_2523_MOESM3_ESM.tif]

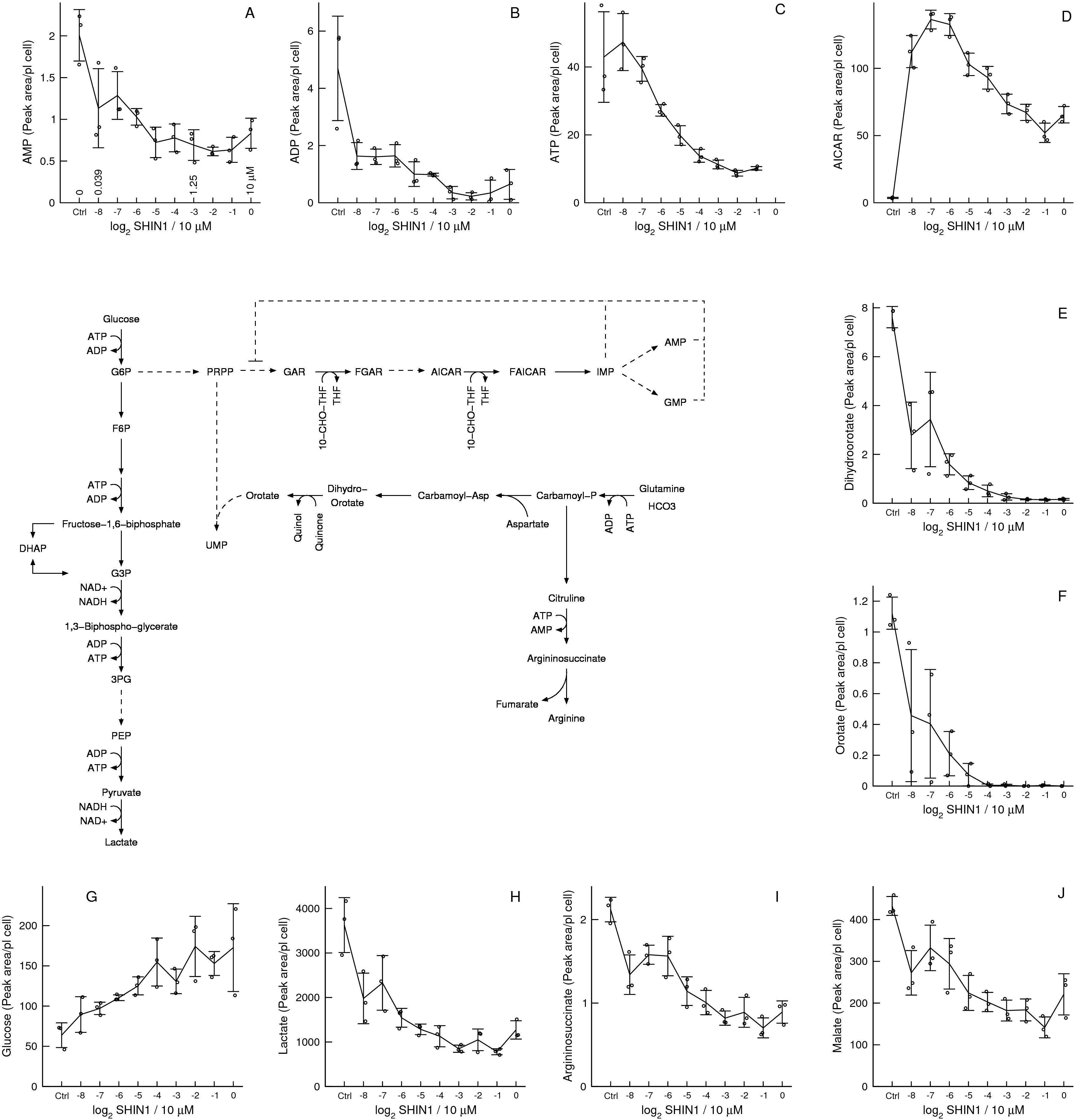

Supplement: Supplementary file 4 — Figure S3 [file 41419_2020_2523_MOESM4_ESM.tif]

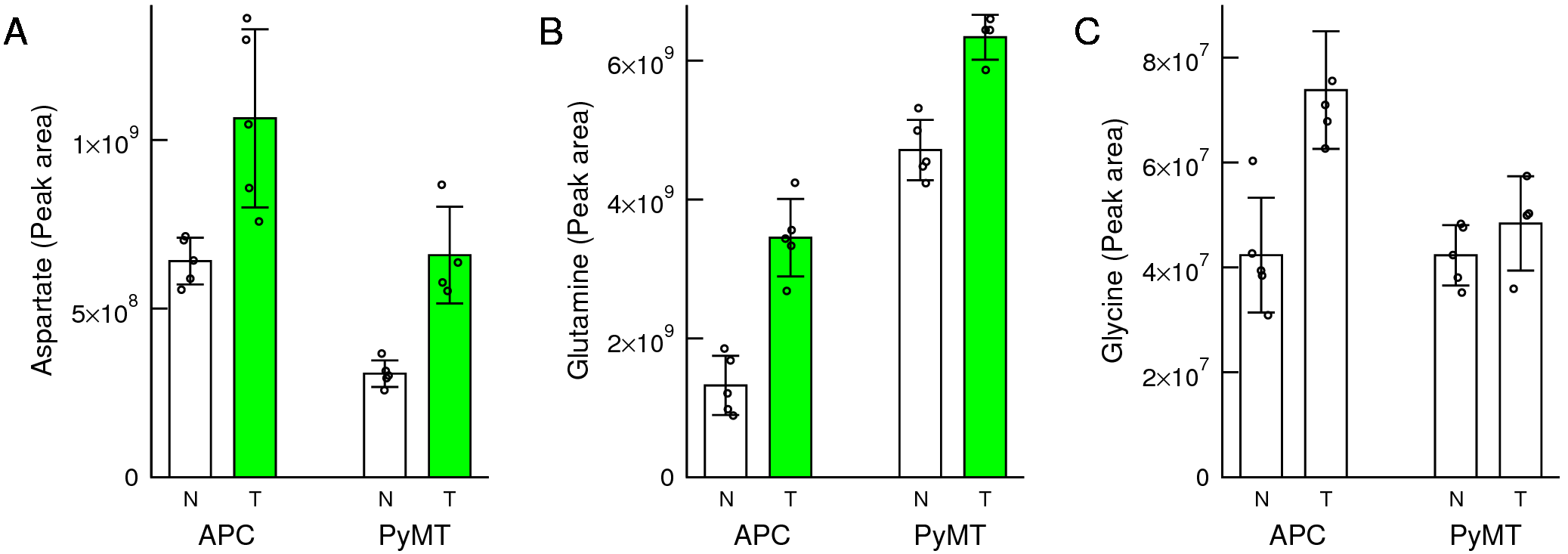

Supplement: Supplementary file 5 — Figure S4 [file 41419_2020_2523_MOESM5_ESM.tif]
