# Supplementary material for: Formate induces a metabolic switch in nucleotide and energy metabolism
Source: Cell Death Dis. 2020 May 4;11(5):310. doi: 10.1038/s41419-020-2523-z (PMC7198490; doi:10.1038/s41419-020-2523-z)
Supplement: Supplementary file 6 — Supplementary text [file 41419_2020_2523_MOESM6_ESM.pdf]

Formate induces a metabolic switch in nucleotide and energy metabolism: Supplementary Text

Kristell Oizel[1], Jacqueline Tait-Mulder[1], Jorge Fernandez-de-Cossio-Diaz[2], Matthias Pietzke[1], Holly Brunton[1], Sandeep Dhayade[1], Sergio Lilla[1], Giovanny Rodriguez Blanco[1], David Sumpton[1], Gillian M. Mackey[1], Karen Blyth[1], Sara Zanivan[1], Johannes Meiser[3], and Alexei Vazquez[1,4]

[1]Cancer Research UK Beatson Institute, Glasgow, UK
[2]Center of Molecular Immunology, Havana, Cuba
[3]Department of Oncology, Luxembourg Institute of Health, L-1526 Luxembourg, Luxembourg
[4]Institute of Cancer Sciences, University of Glasgow, Glasgow, UK

February 20, 2020

1 Mathematical model of formate, purines and energy metabolism

In the mathematical model we write down the equations characterizing the biochemical transformations indicated in Fig. 1A of the main text.

1.1 Formate balance

We assume that formate is produced from the mitochondrial catabolism of serine to formate and formate uptake

$$f_F = f_{SCF} + k_F[For_x] \tag{1}$$

where f_{SCF} is the rate of serine catabolism to formate, k_F is the rate of formate uptake per unit of formate and $[For_x]$ is the concentration of extracellular formate. We assume that the rate of 10-formyl-tetrahydrofolate (CHO-THF) formation from intracellular formate follows a Michaelis-Menten model with respect to the intracellular concentration of formate

$$f_{F,CHO-THF} = h\frac{[For]}{H + [For]} \tag{2}$$

where h is the maximum rate of 10-formyl-tetrahydrofolate synthetase (FTHFS), H is the half-saturation constant of FTHFS for formate and $[For]$ is the intracellular formate concentration. We assume that the rates of formate production is balanced by the rate of FTHFS and formate release

$$f_F = f_{F,CHO-THF} + k_F[For] \tag{3}$$

We solve the equation above for $[For]$ obtaining

$$[For]_\pm = \frac{1}{2}\left(\frac{f_F - h}{k_F} - H \pm \sqrt{\left(\frac{f_F - h}{k_F} - H\right)^2 + 4\frac{f_F}{k_F}H}\right) \tag{4}$$

Since $[For]_- < 0$ the only biologically relevant solution is

$$[For] = \frac{1}{2}\left(\frac{f_F - h}{k_F} - H + \sqrt{\left(\frac{f_F - h}{k_F} - H\right)^2 + 4\frac{f_F}{k_F}H}\right) \tag{5}$$

While the latter equation is convenient for the calculation of formate it does not provide an insight of the possible scenarios. Instead we can solve equation (3) for the extreme cases of f_F. When $f_F \to 0$ we expect $[For] \to 0$, $f_{CHO-THF} \sim h[For]/H$ and the solution to (3) can be approximated by

$$[For]_0 \sim \frac{f_F}{\frac{h}{H} + k_F} \tag{6}$$

In contrast, when $f_F \to \infty$ we expect $[For] \to \infty$, $f_{CHO-THF} \sim h$ and the solution to (3) can be approximated by

$$[For]_\infty \sim \frac{f_F}{k_F} \tag{7}$$

In both limiting cases the formate concentration is proportional to f_F but with different slopes. The ratio

$$\theta = \frac{[For]_\infty}{[For]_0} = \frac{h}{Hk_F} + 1 \tag{8}$$

quantifies the differences between the two limiting cases and quantifies the expected magnitude of the metabolic switch in intracellular formate levels. In mammals the 10-formyltetrahydrofolate synthetase activity is carried on by the tri-functional enzyme MTHFD1. The maximum activity of 10-formyltetrahydrofolaye synthetase (h) will be determined by the levels of MTHFD1, which can change across different cell lines and tissues.

1.2 Adenine nucleotides balance

We assume that the rates of ADP phosphorylation (energy production) and ATP dephosphorylation (energy consumption) are balanced

$$e_g\frac{[ADP]}{E_g + [ADP]} + e_o\frac{[ADP]}{E_o + [ADP]} = a\frac{[ATP]}{A + [ATP]} \tag{9}$$

where e_g and e_o are the maximum rates of ATP production by glycolysis and oxidative phosphorylation, E_g and E_o are the effective half-saturation constants of glycolysis and oxidative phosphorylation, a is the maximum ATPase rate and A its half-saturation constant. The adenine nucleotides are also linked via the adenylate kinase (ADK) equilibrium

$$[AMP][ATP] = K[ADP]^2 \tag{10}$$

where K is the ADK equilibrium constant.

1.3 Purines balance

The interaction between formate and energy metabolism takes place through purine synthesis. We assume that the rate of CHO-THF production by FTHFS and the cytosolic one-carbon metabolism is balanced by the biosynthetic demand of one-carbon units for purine synthesis

$$f_{CHO-THF} = f_{F,CHO-THF} + f_{S,CHO-THF} = \mu\left(2[AMP] + 2[ADP] + 2[ATP] + [RNA] + \frac{5}{4}[DNA]\right) \tag{11}$$

where $f_{S,CHO-THF}$ is the overall rate of cytosolic oxidation of the third carbon of serine to CHO-THF, μ is the proliferation rate and we have assumed a requirement of 2 formate molecules per purine (A and G) and 1 formate molecule per thymidylate (T). We further assume that the proliferation rate is given by the overall rate of growth dependent ATPases, which follow an effective Michaelis-Menten model with respect to the concentration of ATP

$$\mu = \frac{1}{\epsilon}\left(a\frac{[ATP]}{A+[ATP]} - m\right) \tag{12}$$

where ϵ is the energy required to duplicate the cell content and m is the growth independent energy demand of cell maintenance. In a second variation of the model we assume that the proliferation rate is determined by the availability of ADP, the precursor of adenine deoxynucleotides. In this case we assume that the proliferation rate follows an effective Michaelis-Menten law with respect to the concentration of ADP

$$\mu = \frac{1}{\epsilon}\left(d\frac{[ADP]}{D+[ADP]} - m\right) \tag{13}$$

In a third variation of the model we assume that the proliferation rate is constant.

1.4 Working model

Putting together the equations above we obtain our working model linking formate and energy metabolism

$$f_F = f_{SCF} + k_F[For_x] \tag{14}$$

$$[For] = \frac{1}{2}\left(\frac{f_F - h}{k_F} - H + \sqrt{\left(\frac{f_F - h}{k_F} - H\right)^2 + 4\frac{f_F}{k_F}H}\right) \tag{15}$$

$$f_{CHO-THF} = h\frac{[For]}{H+[For]} + f_{S,CHO-THF} \tag{16}$$

$$f_{CHO-THF} = \mu\left(2[AMP] + 2[ADP] + 2[ATP] + [RNA] + \frac{5}{4}[DNA]\right) \tag{17}$$

$$e_g\frac{[ADP]}{E_g+[ADP]} + e_o\frac{[ADP]}{E_o+[ADP]} = a\frac{[ATP]}{A+[ATP]} \tag{18}$$

$$[AMP][ATP] = K[ADP]^2 \tag{19}$$

$$\mu = \frac{1}{\epsilon}\left(a\frac{[ATP]}{A+[ATP]} - m\right) \tag{20}$$

This system of equations can be solved for $[AMP]$, $[ADP]$, $[ATP]$ and $[For]$ as a function of the rate of serine catabolism to formate (f_{SCF}), the cytosolic serine catabolism to CHO-THF ($f_{S,CHO-THF}$) and the extracellular concentration of formate ($[For_x]$). We can also make predictions for the formate release rate, the lactate release rate and the ATP linked respiration rate

$$f_{Formate} = k_F\left([For] - [For_x]\right) \tag{21}$$

$$f_{Lactate} = e_g\frac{[ADP]}{E_g+[ADP]} \tag{22}$$

$$f_{O2,ATP} = \frac{e_o}{2PO}\frac{[ADP]}{E_o+[ADP]} \tag{23}$$

where PO is the P/O ratio.

1.5 Numerical solution

To obtain the plots reported in Fig. 1A we solve the system of equations (14-20) numerically using the octave script provided in this submission, including all parameter estimates and sources.

2 Methematical model of orotate metabolism

We assume that the production and consumption of orotate are balanced resulting in a steady state concentration of orotate. We assume that the orotate production is limited by the ATP dependent activity of carbamoyl-phosphate synthetase and we model its kinetics by a Hill equation with Hill coefficient 2. We assume that the orotate turnover follows a Miachalis-Menten equation with respect to the orotate concentration. Putting all together we obtain the flux balance equation

$$V_1 \frac{[ATP]^2}{K_1 + [ATP]^2} = V_2 \frac{O}{K_2 + [O]} \tag{24}$$

where $[ATP]$ and $[O]$ denote the ATP and orotate concentrations. Solving the latter equation for orotate we obtain

$$[O] = \frac{\theta K_2 [ATP]^2}{K_1 + (1-\theta)[ATP]^2} \tag{25}$$

where

$$\theta = \frac{V_1}{V_2} \tag{26}$$

For $\theta < 1$ this solution is basically a Hill equation with Hill coefficient 2. That is, the concentration of orotate will saturate to a maximum value. In this case we would expect a modest change in the orotate concentration that would depend on the magnitude of θ, the ratio between the maximum activity of synthesis and turnover of orotate. In contrast, for $\theta > 1$ this solution has a divergence when the concentration of ATP approaches $\sqrt{K_1/(\theta - 1)}$.

3 Kinetic model of glycolysis

The computational model used for the simulations of glycolysis is a reduced version of the one in Ref. [2]. It consists of ten ordinary differential equations for the dynamics of the concentrations of the glycolytic intermediates, starting from glucose transport into the cell and ending in the conversion of pyruvate to lactate:

$$\frac{d[glc]}{dt} = J_{glut} - J_{hk} \tag{27}$$

$$\frac{d[g6p]}{dt} = J_{hk} - J_{pgi} \tag{28}$$

$$\frac{d[f6p]}{dt} = J_{pfk} - J_{aldo} \tag{29}$$

$$\frac{d[dhap]}{dt} = J_{aldo} + J_{tpi} \tag{30}$$

$$\frac{d[gap]}{dt} = J_{aldo} - J_{tpi} - J_{gapdh} \tag{31}$$

$$\frac{d[bpg]}{dt} = J_{gapdh} - J_{pgk} \tag{32}$$

$$\frac{d[pg3]}{dt} = J_{pgk} - J_{pgm} \tag{33}$$

$$\frac{d[pg2]}{dt} = J_{pgm} - J_{eno} \quad (34)$$

$$\frac{d[pep]}{dt} = J_{eno} - J_{pk} \quad (35)$$

$$\frac{d[pyr]}{dt} = J_{pk} - J_{ldh} \quad (36)$$

where $J_{reaction}$ denotes a reaction rate and $[metabolite]$ the metabolite concentration.

We list the flux expression and parameter values for all the reactions included in the model as well as the original references. For reversible reactions where both the maximum forward (V_f) and backward (V_b) fluxes appear in the expression (for instance PGI below), the value of V_b was adjusted to obey the Haldane relation [3]:

$$V_b = V_f K_{mb}/(K_{eq} K_{mf}) \quad (37)$$

where K_{mf}, K_{mb} are the reactant constants for the forward and backward directions and K_{eq} the equilibrium constant.

3.1 Glucose uptake

$GLC_{ext} \rightleftharpoons GLC$. The uptake of glucose follows a monosubstrate reversible Michaelis-Menten equation:

$$J_{glut} = V_{max,f} \frac{[glc]_{ext} - [glc]/k_{eq}}{K_{glc,e}(1 + [glc]/K_{glc}) + [glc]_{ext}} \quad (38)$$

where $[glc]_{ext}$ is the concentration of extracellular glucose, $V_{max,f} = 23.03M/min$, $k_{eq} = 1$, $K_{glc} = 0.0093M$ and $K_{glc,e} = 0.01M$ [2, 4].

3.2 Hexokinase

$GLC + ATP \rightleftharpoons G6P + ADP$, random bi-substrate MichaelisMenten:

$$J_{hk} = \frac{\frac{V_{mf}}{K_a K_b}\left(AB - \frac{PQ}{K_{app}}\right)}{1 + \frac{A}{K_a} + \frac{B}{K_b} + \frac{AB}{K_a K_b} + \frac{P}{K_p} + \frac{Q}{K_q} + \frac{PQ}{K_p K_q} + \frac{AQ}{K_a K_q} + \frac{PB}{K_p K_b}} \quad (39)$$

where $A = [glc]$, $B = [atp]$, $P = [g6p]$, $Q = [adp]$, $V_{mf} = 86.85M/min$, $K_a = 0.1mM$, $K_b = 1.1mM$, $K_p = 0.02mM$, $K_q = 3.5mM$, $K_{app} = 651$ [2, 4].

3.3 Phosphoglucoisomerase

$G6P \rightleftharpoons F6P$. Monoreactant reversible equation with competitive inhibition by E4P, 6PG, and FBP:

$$J_{pgi} = \frac{V_{mf}\frac{[g6p]}{K_{g6p}} - V_{mr}\frac{[f6p]}{K_{f6p}}}{1 + \frac{[g6p]}{K_{g6p}} + \frac{[f6p]}{K_{f6p}} + \frac{[e4p]}{K_{ery4p}} + \frac{[f16p]}{K_{fbp}} + \frac{[pgn]}{K_{pg}}} \quad (40)$$

where $V_{mr} = V_{mf} K_{f6p}/(K_{eq} K_{g6p})$, $K_{g6p} = 0.4mM$, $K_{f6p} = 0.05mM$, $K_{ery4p} = 0.001mM$, $K_{fbp} = 0.06mM$, $K_{pg} = 0.015mM$, $K_{eq} = 55.6mM$ [4].

3.4 Phosphofructokinase

$F6P + ATP \rightleftharpoons F16P + ADP$. Flux expression from [4].

$$J_{pfk} = V_m \frac{\frac{[atp]}{K_{atp}}}{1+\frac{[atp]}{K_{atp}}} \frac{1+\frac{\beta}{\alpha}\frac{[f26bp]}{K_{a,f26bp}}}{1+\frac{1}{\alpha}\frac{[f26bp]}{K_{a,f26bp}}} \frac{\frac{[f6p]\left(1+\frac{1}{\alpha}\frac{[f26bp]}{K_{a,f26bp}}\right)}{K_{f6p}\left(1+\frac{[f26bp]}{K_{a,f26bp}}\right)}\left(1+\frac{[f6p]\left(1+\frac{1}{\alpha}\frac{[f26bp]}{K_{a,f26bp}}\right)}{K_{f6p}\left(1+\frac{[f26bp]}{K_{a,f26bp}}\right)}\right)^3}{\frac{L\left(1+\frac{[cit]}{K_{i,cit}}\right)^4\left(1+\frac{[atp]}{K_{i,atp}}\right)^4}{\left(1+\frac{[f26bp]}{K_{a,f26bp}}\right)^4} + \left(1+\frac{[f6p]\left(1+\frac{1}{\alpha}\frac{[f26bp]}{K_{a,f26bp}}\right)}{K_{f6p}\left(1+\frac{[f26bp]}{K_{a,f26bp}}\right)}\right)^4} - V_m \frac{\frac{[adp][fbp]}{K_{adp}K_{fbp}K_{eq}}}{1+\frac{[adp]}{K_{adp}}+\frac{[fbp]}{K_{fbp}}+\frac{[adp][fbp]}{K_{adp}K_{fbp}}} \tag{41}$$

where $V_m = 107.6 M/s$, $K_{atp} = 0.021 mM$, $\beta = 0.98$, $\alpha = 0.32$, $K_{f26bp} = 0.00084 mM$, $K_{f6p} = 1 mM$, $L = 4.1 mM$, $K_{cit} = 6.8 mM$, $K_{i,atp} = 20 mM$, $K_{adp} = 5 mM$, $K_{fbp} = 5 mM$, $K_{app} = 247$ [4].

3.5 Fructose bisphosphate aldolase

$F16P \rightleftharpoons GAP + DHAP$. Flux expression from [4].

$$J_{aldo} = \frac{V_{mf}\frac{A}{K_{fbp}} - V_{mr}\frac{PQ}{K_{dhap}K_{g3p}}}{1+\frac{A}{K_{fbp}}+\frac{P}{K_{dhap}}+\frac{Q}{K_{g3p}}+\frac{PQ}{K_{dhap}K_{g3p}}} \tag{42}$$

where $V_{mf} = 14.63 M/s$, $A = [f16p]$, $P = [dhap]$, $Q = [gap]$, $K_{fbp} = 0.009 mM$, $K_{dhap} = 0.08 mM$, $K_{g3p} = 0.16 mM$, $K_{eq} = 0.0018$ [4].

3.6 Triose-phosphate isomerase

$GAP \rightleftharpoons DHAP$. Flux expression from [4].

$$J_{tpi} = \frac{V_f\frac{[gap]}{K_{ms}} - V_r\frac{[dhap]}{K_{mp}}}{1+\frac{[gap]}{K_{ms}}+\frac{[dhap]}{K_{mp}}} \tag{43}$$

where $V_r = V_f(K_{mp}/K_{ms})/K_{eq}$, $V_f = 5.976 M/s$, $K_{ms} = 0.51 mM$, $K_{mp} = 1.6 mM$ and $K_{eq} = 0.381$ [4].

3.7 Glyceraldehyde-3-phosphate dehydrogenase

$GAP + NAD + Pi \rightleftharpoons BPG + NADH + H$. Flux expression from [4].

$$J_{gapdh} = \frac{V_{mf}\frac{ABC}{K_{nad}K_{g3p}K_p} - V_{mr}\frac{PQ}{K_{dgp}K_{nadh}}}{1+\frac{A}{K_{nad}}+\frac{AB}{K_{nad}K_{g3p}}+\frac{ABC}{K_{nad}K_{g3p}K_p}+\frac{PQ}{K_{dpg}K_{nadh}}+\frac{Q}{K_{nadh}}} \tag{44}$$

where $A = [nad]$, $B = [gap]$, $C = [pi]$, $P = [bpg]$, $Q = [nadh]$, $V_{mr} = (V_{mf}/K_{eq})K_{dpg}K_{nadh}/(K_{g3p}K_{nad}K_p)$, $V_{mf} = 109.1 M/s$, $K_{nad} = 0.09 mM$, $K_{g3p} = 0.19 mM$, $K_p = 29 mM$, $K_{dpg} = 0.022 mM$, $K_{nadh} = 0.01 mM$, $K_{eq} = 0.3574$ [4].

3.8 Phosphoglycerate kinase

$BPG + ADP \rightleftharpoons PG3 + ATP$. Flux expression from [4].

$$J_{pgk} = \frac{V_{mf}\frac{AB}{\alpha K_a K_b} - V_{mr}\frac{PQ}{\beta K_p K_q}}{1 + \frac{A}{K_a} + \frac{B}{K_b} + \frac{AB}{\alpha K_a K_b} + \frac{PQ}{\beta K_p K_q} + \frac{P}{K_p} + \frac{Q}{K_q}} \tag{45}$$

where $A = [bpg]$, $B = [adp]$, $P = [pg3]$, $Q = [atp]$, $V_{mr} = V_{mf}K_pK_q/(K_{eq}K_aK_b)$, $\alpha = 1$, $K_a = 0.079mM$, $K_b = 0.04mM$, $\beta = 1$, $K_p = 0.13mM$, $K_q = 0.27mM$, $K_{eq} = 11.369$ [4].

3.9 Phosphoglycerate mutase

$PG3 \rightleftharpoons PG2$. Flux expression from [4].

$$J_{pgm} = \frac{V_{mf}\frac{[pg3]}{K_{ms}} - V_{mr}\frac{[pg2]}{K_{mp}}}{1 + \frac{[pg3]}{K_{ms}} + \frac{[pg2]}{K_{mp}}} \tag{46}$$

where $V_{mr} = V_{mf}K_{mp}/(K_{eq}K_{ms})$, $K_{ms} = 0.19mM$, $K_{mp} = 0.12mM$, $K_{eq} = 1.6491$ [4].

3.10 Enolase

$PG2 \rightleftharpoons PEP$, monosubstrate simple reversible Michaelis-Menten kinetics,

$$J_{eno} = \frac{V_{mf}\frac{[pg2]}{K_{ms}} - V_{mr}\frac{[pep]}{K_{mp}}}{1 + \frac{[pg2]}{K_{ms}} + \frac{[pep]}{K_{mp}}} \tag{47}$$

where $V_{mr} = V_{mf}K_{mp}/(K_{eq}K_{ms})$, $K_{ms} = 0.038mM$, $K_{mp} = 0.06mM$, $K_{eq} = 1.4127$ [4].

3.11 Pyruvate kinase

$PEP + ADP \rightleftharpoons PYR + ATP$. Flux expression from [4].

$$J_{pk} = V_m \left\{ \frac{\frac{[adp]}{K_{adp}}}{1 + \frac{[adp]}{K_{adp}}} \frac{\frac{[pep]}{K_{pep}}\left(1 + \frac{[pep]}{K_{pep}}\right)^3}{\frac{L\left(1+\frac{[atp]}{K_{i,atp}}\right)^4}{\left(1+\frac{[fbp]}{K_{a,fbp}}\right)^4} + \left(1 + \frac{[pep]}{K_{pep}}\right)^4} - \frac{\frac{[atp][pyr]}{K_{atp}K_{pyr}K_{eq}}}{1 + \frac{[atp]}{K_{atp}} + \frac{[pyr]}{K_{pyr}} + \frac{[atp]}{K_{atp}}\frac{[pyr]}{K_{pyr}}} \right\} \tag{48}$$

where $V_m = 27.81M/s$, $K_{adp} = 0.4mM$, $K_{pep} = 0.014mM$, $L = 1$, $K_{i,atp} = 2.5mM$, $K_{fbp} = 0.0004mM$, $K_{atp} = 0.86mM$, $K_{pyr} = 10mM$, $K_{app} = 195172$ [4].

3.12 Lactate dehydrogenase

$PYR + NADH \rightleftharpoons LAC + NAD$. Flux expression from [4].

$$J_{ldh} = \frac{V_{mf}\frac{AB}{\alpha K_a K_b} - V_{mr}\frac{PQ}{\beta K_p K_q}}{1 + \frac{A}{K_a} + \frac{B}{K_b} + \frac{AB}{\alpha K_a K_b} + \frac{P}{K_p} + \frac{Q}{K_q} + \frac{PQ}{\beta K_p K_q}} \tag{49}$$

where $A = [nadh]$, $B = [pyr]$, $P = [lac]$, $Q = [nad]$, $V_{mr} = V_{mf}K_pK_q/(K_{eq}K_aK_b)$, $\alpha = 1$, $K_a = 0.002mM$, $K_b = 0.3mM$, $\beta = 1$, $K_p = 4.7mM$, $K_q = 0.07mM$, $K_{eq} = 3.4525 \times 10^3$ [4].

3.13 Simulations

The concentrations of extracellular glucose, lactate, ATP, ADP, AMP, phosphate, NADH and NAD^+ were held fixed during the simulation. The original model [2] includes reactions of the pentose phosphate pathway and generic ATPase and dehydrogenase reactions that represent demands of ATP and NADH by other cellular processes. These reactions were not considered in our simulations since we are interested only in the flux through glycolysis and because the concentrations of cofactors are held constant. To determine what metabolite concentrations control the glycolytic flux, the fixed concentrations of ADP, AMP, NADH and NAD^+ were sampled at random within a physiological range (see Table below). For each set of concentrations the resulting flux of glycolysis at steady state was recorded.

Metabolite	Min. concentration (M)	Max. concentration (M)
ATP	0.001	0.009
ADP	3×10^{-5}	0.0015
AMP	0.00156	0.00622
NAD	0.00065	0.0026
NADH	0.00021	0.00086

The code was written in Julia [5]. We used the DifferentialEquations.jl package [6] to perform the simulations until a steady state was reached.

4 Kinetic model of oxidative phosphorylation

The kinetic model of oxidative phosphorylation was based on Ref.[7]. It consists of a system of 18 differential equations describing metabolite dynamics in the mitochondrial matrix, the intermembrane space, and exchanges with the cytosol. We simulated the model as described in the original reference, with the only change of setting the relative volume of the cytoplasm to infinity to simulate constant concentrations of metabolites outside the mitochondria. The concentrations of cytosolic ATP, ADP, and AMP were randomly sampled in the same range as in the glycolysis simulations (Table above). From the simulations it was concluded that the rate of ATP production by ATP synthase follows approximately a Michaelis-Menten equation with respect to the ADP concentration (Fig. 1C in teh main text), with a half-saturation constant of 0.12 mM ADP.

References

[1] Fernandez-de-Cossio-Diaz, J. & Vazquez, A. Limits of aerobic metabolism in cancer cells. *Scientific Reports* **7**, 13488 (2017).

[2] Mosca, E. *et al.* Computational modeling of the metabolic states regulated by the kinase akt. *Frontiers in physiology* **3**, 418 (2012).

[3] Klipp, E., Liebermeister, W., Wierling, C. & Kowald, A. *Systems Biology: A Textbook* (Wiley-VCH Verlag GmbH & Co. KGaA, Weinheim, 2016), second, completely revised and enlarged edition edn.

[4] Marín-Hernández, A. *et al.* Modeling cancer glycolysis. *Biochimica et Biophysica Acta (BBA)-Bioenergetics* **1807**, 755–767 (2011).

[5] Bezanson, J., Edelman, A., Karpinski, S. & Shah, V. B. Julia: A fresh approach to numerical computing. *SIAM review* **59**, 65–98 (2017).

[6] Rackauckas, C. & Nie, Q. Differentialequations. jl–a performant and feature-rich ecosystem for solving differential equations in julia. *Journal of Open Research Software* **5** (2017).

[7] Heiske, M., Letellier, T. & Klipp, E. Comprehensive mathematical model of oxidative phosphorylation valid for physiological and pathological conditions. *The FEBS Journal* **284**, 2802–2828 (2017).
